# ﻿Taxonomic insights into *Panopeuslacustris* Desbonne (Crustacea, Decapoda, Brachyura) based on morphological and molecular data

**DOI:** 10.3897/zookeys.1179.105734

**Published:** 2023-09-08

**Authors:** Jani Jarquín-González, Martha Valdez-Moreno, Rigoberto Rosas-Luis

**Affiliations:** 1 Tecnológico Nacional de México/I.T. de Chetumal, Av. Insurgentes 330, Chetumal 77013, Quintana Roo, Mexico Tecnológico Nacional de México/I.T. de Chetumal Chetumal Mexico; 2 El Colegio de la Frontera Sur, Av. Centenario km 5.5, Chetumal 77014, Quintana Roo, Mexico El Colegio de la Frontera Sur Chetumal Mexico; 3 CONAHCYT-Tecnológico Nacional de México/I.T. de Chetumal, Av. Insurgentes 330, Chetumal 77013, Quintana Roo, Mexico CONAHCYT-Tecnológico Nacional de México/I.T. de Chetumal Chetumal Mexico

**Keywords:** COI gen, Mexican Caribbean, Morphology, Taxonomy, True crabs

## Abstract

The genus *Panopeus* belongs to the family Panopeidae and comprises a group of brachyuran crabs native to the American continent (except for *P.africanus*). However, taxonomic problems related to the presence of cryptic species have made it difficult to recognize the species and advance the biological knowledge of this group. Herein, a detailed description based on morphological and molecular data is provided for the species *P.lacustris* for the first time. Additionally, new morphological characters are proposed for the discrimination of the species. It is strongly suggested to increase the knowledge of the gene pool of the group, explore new morphological characters, and update the species descriptions to advance the group’s knowledge. This proposal will serve as a reference for future studies aimed at clarifying the taxonomic, conservation and ecological status of the species of *Panopeus*.

## ﻿Introduction

The genus *Panopeus* H. Milne Edwards, 1834 belongs to the family Panopeidae Ortmann, 1893 and comprises a group of brachyuran crabs native to the American continent (except for *P.africanus* A. Milne-Edwards, 1867) and which are among the most abundant and conspicuous invertebrates of marine and coastal habitats (Benedict and Rathbun 1891; Schubart et al. 2000). They are mainly associated with soft sediment bottoms with seagrasses, mangrove forests, and oyster beds. However, according to Williams (1983), the exact ecological roles of *Panopeus* species could be uncertain because of imprecise taxonomic identifications.

Morphologically, they are distinguished by having five lateral teeth in the carapace, of which the first two can be partially fused; unequal chelipeds; and a prominent basal tooth on the dactylus in the major chela (Rathbun 1930; Williams 1983). But taxonomic and morphological problems related to the presence of cryptic species have made it difficult to recognize the species and advance the biological knowledge of this group (Williams 1983; Schubart et al. 2000). Martin and Abele (1986) point out that the morphology and ornamentation of the male gonopod are valid taxonomic characters in the differentiation of genera and species since they do not reflect adaptations to the habitat or types of feeding. According to Thoma et al. (2014), additional studies are necessary to include new morphological characters and genetic data to clarify the taxonomic status and the evolutionary relationships of *Panopeus* species. This method has been widely used to verify and delimit closely related species and groups of marine organisms, including decapods (Meyer et al. 2013; Magalhães et al. 2016; Yuan et al. 2022). According to Matzen da Silva et al. (2011), in decapods the maximum distance value of interspecific divergence, using COI gene, corresponds to 4.6% approximately.

*Panopeus* contains 16 species distributed in the Eastern Pacific, Western Atlantic, and Eastern Atlantic (Table 1). Eleven species were described between 1834 and 1880, and five species between 1915 and 1983. After this, the genus has not been revised and updated, and no new species have been recognised. A detailed description of *P.lacustris* is presented, and distributional and genetic information is added.

**Table 1.** Species of the genus *Panopeus* including taxonomic (authorities, type locality, type status and museum respository), ecological (general distribution, type of habitat) and molecular data (BOLDSYSTEMS accession numbers available in dx.doi.org/10.5883/DS-PANOPE01).

Species	Type Locality	Type Material	Distribution	Habitat	Bin
*Panopeusafricanus* A. Milne-Edwards, 1867	Gabon and Angola, Africa	Syntype USNM 20263	Eastern Atlantic—Portugal and Spain to Angola (Manning and Holthuis 1981)	Oyster cultch, intertidal, lagoon, river’s mouth (Manning and Holthuis 1981)	BOLD:ADK5484
*Panopeusamericanus* de Saussure, 1857	Guadeloupe, Antilles	Syntypes MHNG-ARTO-16417 to MHNG-ARTO-16422	Western Atlantic—USA (Florida), Gulf of Mexico, Antilles, north of South America, and Brazil (Almeida et al. 2010; Hollier 2018; Mantelatto et al. 2020)	Under rocks, on mud beaches and in mangroves, on sand, mud, and shell bottoms. 0–25 m (Almeida et al. 2010)	BOLD:ADU0973, BOLD:ADX1970
*Panopeusaustrobesus* Williams, 1983	Paranagua, Brazil	Holotype USNM 59462	Western Atlantic—Brazil (Mantelatto et al. 2020)	Sandy mud flats, under scattered rocks (Williams 1983)	BOLD:ADT6599
*Panopeusboekei* Rathbun, 1915	Tumble-Down-Dick Bay, Netherlands Antilles	Syntype RMNH.CRUS.D.2226	Western Atlantic—Antilles (St Thomas, St Eustatius, Bonaire, Curaçao) (Poupin 2018)	Lagoon, mangrove, stony bottom, sand, among algae. 0–15 m (Rathbun 1930; Poupin 2018)	-
*Panopeuschilensis* H. Milne Edwards & Lucas, 1843	Chile	Syntype USNM 20264	Eastern Pacific—From Mexico (Sonora) to Chile (Hendrickx 1995)	Among rocks. Intertidal (Rathbun 1930)	BOLD:ADT4346
*Panopeusconvexus* A. Milne-Edwards, 1880	Chile	Type probably in MNHN	Eastern Pacific—Chile (Rathbun 1930)	-	-
*Panopeusdiversus* Rathbun, 1933	San Felipe, Baja California, Mexico	Holotype USNM 67570	Eastern Pacific—Gulf of California (Hendrickx 1995)	-	-
*Panopeusharttii* Smith, 1869	Abrolhos Reefs, Brazil	Syntype MCZH CRU-4806	Western Atlantic—USA (Florida), Southern Mexican Caribbean, Cuba, Virgin Islands, islands of the Caribbean Arc, Aruba, and Brazil. Central Atlantic—Ascension Island (Jarquín-González et al. 2022).	Coralline reefs, rocky bottoms, dead coral. On calcareous algae blocks, and on *Halimeda* sp. 0–25 m (Jarquín-González et al. 2022)	BOLD:AAX2632
*Panopeusherbstii* H. Milne Edwards, 1834	North American coast	Type probably in MNHN	Western Atlantic—USA (Massachusetts to Florida), Gulf of Mexico, Antilles, Caribbean Sea, north of South America, and Brazil (Mantelatto et al. 2020)	Mangrove roots, sponges, coralline reefs. In muddy, shells or rocky bottom. In oyster beds. 0–22 m (Williams 1983)	BOLD:ACC3621
*Panopeuslacustris* Desbonne in Desbonne & Schramm, 1867	Guadeloupe, Antilles	Unknown	Western Atlantic—USA (Georgia to Florida), Bermuda, Bahamas, Gulf of Mexico, Mexican Caribbean, Panama, Colombia, Cuba, Jamaica, Puerto Rico, Virgin Islands, islands of the Caribbean Arc, islands off Venezuela, and Brazil. Western Pacific: Hawaii (Abele and Kim 1986; Jarquín-González et al. 2022)	Sandy and rocky beach, coralline reef (*Porites* sp.), and sabellarid reef. In Coralline rocks in *Thalassiatestudinum* beds. In sponge on mangrove. 0–2 m (Jarquín-González et al. 2022)	BOLD:ACU0442
*Panopeusmeridionalis* Williams, 1983	Punta Carretas, Uruguay	Holotype USNM 99846	Western Atlantic— Uruguay and Argentina (Spivak et al. 2019)	Rocky coast (Williams 1983)	-
*Panopeusobesus* Smith, 1869	Egmont Key, Florida, USA	Syntype YPM IZ 000901.CRB	Western Atlantic—USA (North Carolina to Florida), and Gulf of Mexico (Williams 1983)	Mangrove roots. Marsh edge, shallow intertidal, and subtidal waters (Williams 1983)	BOLD:ACC3621
*Panopeusoccidentalis* de Saussure, 1857	Guadeloupe, Antilles	Syntypes MHNG-ARTO-16427-MHNG-ARTO-16429	Western Atlantic: from USA (North Carolina) to Brazil (Hollier 2018; Jarquín-González et al. 2022)	Sandy, rocky and shells bottoms. Among rocks, mangrove roots, sponges, ascidians, and macroalgae. 1–20 m depth (Jarquín-González et al. 2022)	BOLD:AAX2632
*Panopeuspurpureus* Lockington, 1877	Magdalena Bay, Baja California, Mexico	Unknown	Eastern Pacific—From Mexico (Baja California) to Peru (Hendrickx 1995; Vargas and Cortés 2019)	Rocky intertidal zone, sandy beach, estuary, mangrove, coralline reef (Vargas and Cortés 2019)	BOLD:ADT4345
*Panopeusrugosus* A. Milne-Edwards, 1880	Bahia, Brazil	Type probably in MNHN	Western Atlantic—Florida, Gulf of Mexico, Central America, West Indies, northern South America, and Brazil. Eastern Pacific—Panama Canal (Hendrickx 1995; Almeida et al. 2010)	Estuaries, under tree trunks, rocks, and rubble, inside decaying tree trunks Also found in fresh water. In the intertidal and shallow subtidal (Almeida et al. 2010)	BOLD:ADT6599
*Panopeussimpsoni* Rathbun, 1930	Saint George’s Sound, Florida, USA	Holotype USNM 56382	Western Atlantic—Gulf of Mexico and Southern Mexican Caribbean (Williams 1983; Jarquín-González et al. 2022)	Shallow intertidal and subtidal waters (Abele and Kim 1986)	BOLD:ACC3621

## ﻿Material and methods

**Figure 1.**
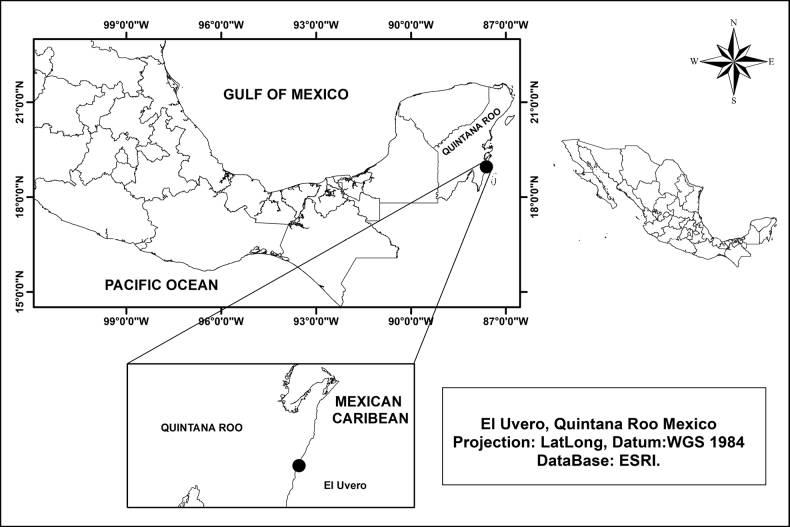
Location of El Uvero, in the State of Quintana Roo, on the Mexican Caribbean.

Specimens identified as *Panopeus* sp. from El Uvero, 18°57.240'N, 87°36.900'W, Quintana Roo, Mexico (Fig. 1), and deposited in the Reference Collection of Benthos (ECOSUR) of El Colegio de la Frontera Sur, Chetumal, Mexico were reviewed.

Morphological characteristics were observed using Zeiss Stemi DV4 stereoscopic microscope. For structures such as gonopods and maxillipeds, the dissection of appendages was generally performed on the right side of the body; the pieces were mounted in glycerol and sealed with transparent nail varnish. Photographs were taken using an Olympus SZX10 stereoscopic microscope, a Martin Microscope Company MDSLR-BX camera-microscope adapter, and a Canon EOS Rebel T3i digital reflex camera. Between 20 and 50 photos were taken of each specimen and its structures. Subsequently, focus stacking was performed with Helicon Focus v. 8.1.0 software. The morphological identification was based on the works of Rathbun (1930) and Williams (1983), while the morphological terminology followed that used by Davie et al. (2015).

Molecular analyses were performed to differentiate the *Panopeus* species and recognize their genetic divergence values. A small piece of muscle (1–3 mm^3^) was used to get the genomic DNA. A lysis buffer was used to digest the sample’s tissue with proteinase K, and the sample was digested in an oven for 12 h at 56 °C. The extraction was done through a 1.0 mm PALL glass fiber plate (Ivanova et al. 2006).

Cytochrome oxidase I (COI) gene segment with an approximate length of 650 bp (Hebert et al. 2003) was amplified using the zooplankton primers (Prosser et al. 2013). DNA barcoding was conducted at Macrogen (Seoul, Republic of Korea). Sequence data, electropherograms, trace files, primer details, photographs, life stages, and collection localities for specimens using in the molecular analysis are available at BOLDSYSTEMS https://www.boldsystems.org within the dataset *Panopeus* from the Southern Mexican Caribbean and other regions of the Western Atlantic and Central Pacific (DS-PANOPE01) (https://www.doi.org/10.5883/DS-PANOPE01, accessed on July 13, 2023).

All the sequences obtained were compared with COI sequences previously published using the specimen identification tool in the Barcode of Life Data System (BOLD) (Sarmiento-Camacho and Valdez-Moreno 2018). Similarity values >98% were considered for all identified species, which confirmed their placement under different Barcode index numbers (BINs) (Ratnasingham and Hebert 2013). The criteria to assign taxonomic level identification using BOLD was a similarity value >99% (Sarmiento-Camacho and Valdez-Moreno 2018). To elaborate the ID tree, the COI gene sequences obtained in this work were used, as well as the sequences available in BOLDSYSTEMS under the following criteria: no taxonomic identification conflict, having geographical coordinates, and having high quality sequences.

All sequences were aligned using the ClustalW method (Thompson et al. 1994); the best substitution model was determined according to the lowest Bayesian information criterion score. As a result, a Tamura 3-parameter was used, using a discrete Gamma distribution (+G) with five rate categories as a model to construct a tree using the maximum likelihood analysis. The Kimura 2-parameter model was used to calculate the genetic divergences between the species (Kimura 1980). All studies used MEGA 11 software (Tamura et al. 2021).

## ﻿Results

**Order Decapoda Latreille, 1802**

**Infraorder Brachyura Latreille, 1802**

**Genus *Panopeus* H. Milne Edwards, 1834**

### ﻿ Panopeus lacustris

Taxon classificationAnimaliaDecapodaPanopeidae

Desbonne in Desbonne & Schramm, 1867

8D40991B-3714-5974-9BA1-24EC7AD4E2E9

Figs 2 , 3 , 4 , 5

#### Material examined.

Mexico • non-ovigerous female, 28.9 mm; Mexico, Quintana Roo, El Uvero; 18°57.240'N, 87°36.900'W; depth 0.5 m; 28 Jan. 2021; collectors Víctor Conde, Astrid Te leg.; in coralline rocks under *Thalassiatestudinum*; ECOSUR-C1117 • non-ovigerous female, 9.5 mm; same collection data as for preceding; ECOSUR-C1195 • one male, 19.3 mm; same collection data as for preceding; ECOSUR-C1196 • one male, 12.5 mm; same collection data as for preceding; ECOSUR-C1197.

#### Diagnosis

**(emended for Williams, 1983).** Carapace smooth or with a few setae, with transverse lines of granules, coarse granules on ocular and hepatic regions and along anterolateral margins. With five lateral teeth, the first two coalesced, separated by a shallow rounded notch (variable in depth). The second tooth is broader than the first, but the tips are almost equally prominent (variable in form). Third, fourth and fifth lateral teeth with prominent or semi-prominent acute tips. Third, maxilliped with one red or orange spot on the ventral surface side of the ischium. Cheliped with two distal spines in merus. The palm of chelipeds with a reticulated pattern of small purple granules. The reticulate pattern of color on the outer surface of the palm is continuous or not on its lower half. With random dark spots below the midline of the palm, ventral side of the palm, and ventral side of the cheliped merus. Dactylus of cheliped with a prominent proximal tooth. Pereopods are setose. **Female**: Abdomen setose, with six pereonites. **Male**: Abdomen with five pereonites. Gonopod 1 has two distal rows of conic spines that increase in thickness as they approach the anterior part of the outer margin, with six subdistal slender spinules on the inner margin.

#### Description.

**Figure 2.**
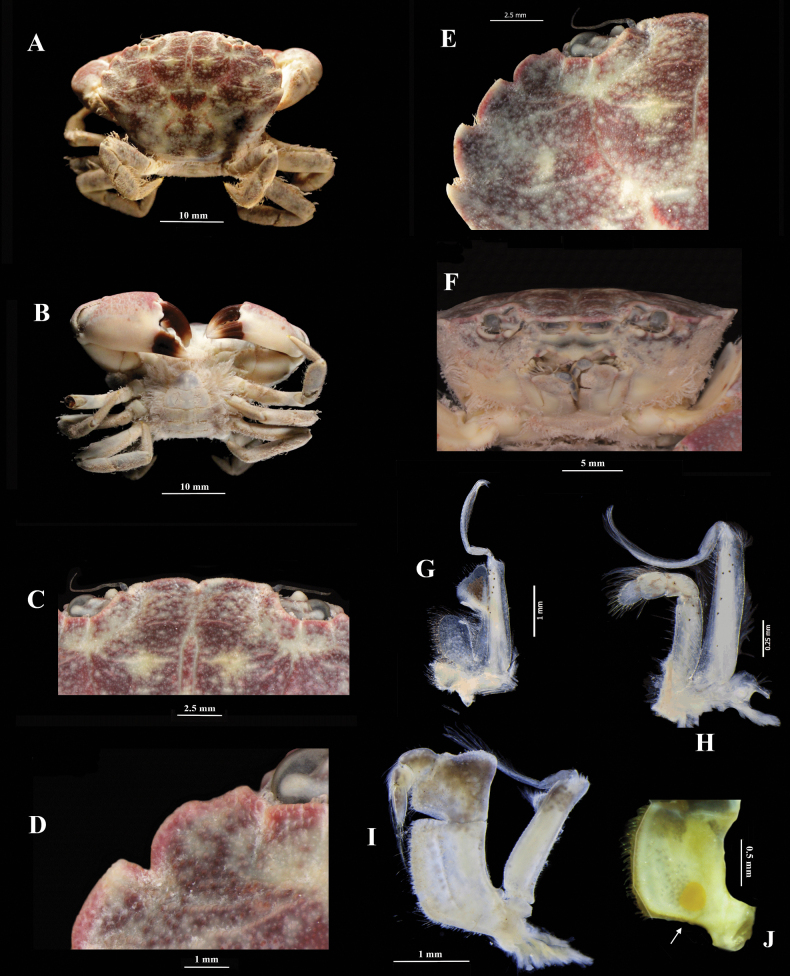
*Panopeuslacustris*. ECOSUR-C1117, non-ovigerous female, 28.9 mm **A** body, dorsal view **B** body, ventral view **C** frontal margin **D** first two coalescent teeth **E** lateral teeth of the carapace **F** frontal view. ECOSUR C1195, non-ovigerous female, 9.5 mm **G** maxilliped **H** maxilliped 2 **I** maxilliped 3 **J** spot on isquium of maxilliped 3.

**Non-ovigerous female**, ECOSUR-C1117, 28.9 mm (Fig. 2A, B). Carapace reddish-brown with creamy-white areas, smooth, surface finely granulate, hexagonal, convex, feebly areolated, with some lines of granules on hepatic and epigastric regions.

The frontal margin is sinuous and granulose, with a median closed fissure and rounded outer angles. First and second lateral teeth coalescent, separated by a shallow rounded notch. The first tooth is small, triangular, and has a prominent tip. The second tooth is broader than the first, convex, with a semi-prominent acute tip. The third tooth is convex, not outstanding, with semi-prominent acute tip. The fourth tooth is convex and outstanding, with a prominent acute tip. The fifth tooth is smaller than the fourth, outstanding spiniform (Fig. 2C–E).

Orbits suboval, wide; orbital region granular; dorsal inner orbital angle separated from the front by a shallow notch. Eyestalk stout, covered with fine granules. Antennular fossettes (Fig. 2F) are subrectangular and flattened; antennules are stout, folding transversely. The basal segment of the antenna is subrectangular; the flagellum is slender, tip reaches an exorbital angle. Epistome broad at base and pointed distally. Posterior margin of endostome with oblique ridges.

First and second maxillipeds with a few monochromatic chromatophores (Fig. 2G, H). The third maxilliped (Fig. 2I, J) completely covers the buccal orifice. Ischium subrectangular, granular, with a medial groove on dorsal surface; inner margin crenulated and setose; outer margin with rounded proximal projection; with one proximal orange spot on the ventral surface. Merus subquadrate, granular. Exopod narrow, longer than broad; inner margin with subdistal projection rounded and setose; outer margin with short distal setae.

Abdomen setose, with all pleonites well defined; first, second and third pleonite longer than broad fourth and fifth pleonites similar, broader than long; sixth pleonite is the largest, sinuous. Telson is subtriangular, broad, with rounded angles and a similar length to the sixth pleonite.

**Figure 3.**
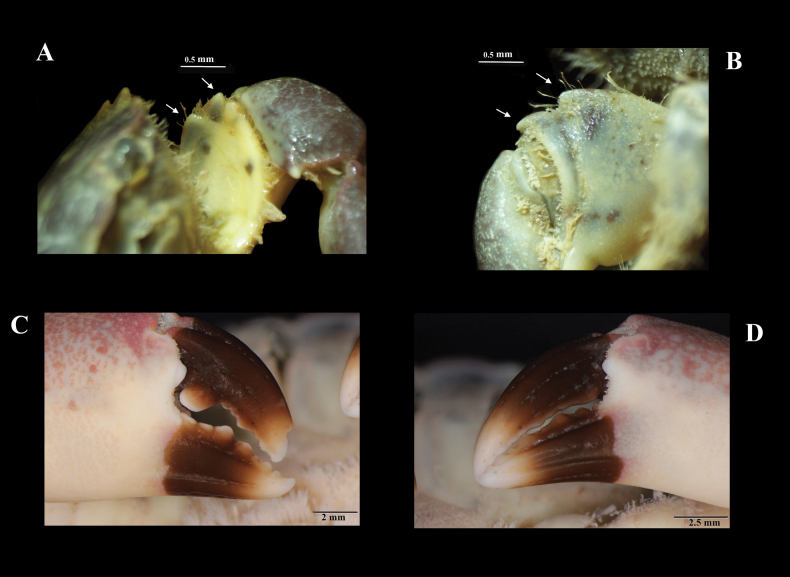
*Panopeuslacustris*. ECOSUR-C1117, non-ovigerous female, 28.9 mm **A, B** spots and spines in merus of chelipeds **C** major chela **D** minor chela.

Chelipeds are unequal, robust, and finely granulate. Merus is broader than carpus, with two distal spines, no setose, and few dark spots ventrally (Fig. 3A, B). Carpus without groove parallel to the distal margin; reticulated pattern present only dorsolaterally; with one pointed tooth on the inner side. Palm with subdistal broad and triangular lobe near dactylus articulation; the reticulate pattern of color present dorsolaterally and does not continue on its lower half; with random dark spots below the midline of the palm, also present on its ventral side. The dark color of pollex does not continue on the palm. Major chela (Fig. 3C), pollex with seven teeth; with cusps raised above a straight line drawn between the angle at the juncture of a finger with anterior margin of palm and tip of the finger; first four teeth very broad; first and second teeth flattened, not separated, with blunt cusp probably due to wear; the third tooth is the largest of all, with a pointed cusp; fourth article broader than following teeth, with blunt cusp; fifth and seventh teeth smaller than sixth tooth, with blunt cusp each one. Dactylus of major chela, with one proximal tooth, prominent, longer than broad, with blunt cusp; with two reduced teeth; with one medial pointed process.

Minor chela (Fig. 3D) with a few small proximal teeth on the inner margin of the pollex. Pollex and dactylus of both chelipeds with white tips.

Ambulatory legs all setose dorsodistally; with dactylus small, broad, and orange tip. Second, third, and fourth pereopods with merus shorter than carpus; carpus inflated, longer than broad; propodus longer than merus, less inflated than carpus. Fifth pereopod is shorter than all, with merus and carpus of similar width; with propodus as long as merus.

**Figure 4.**
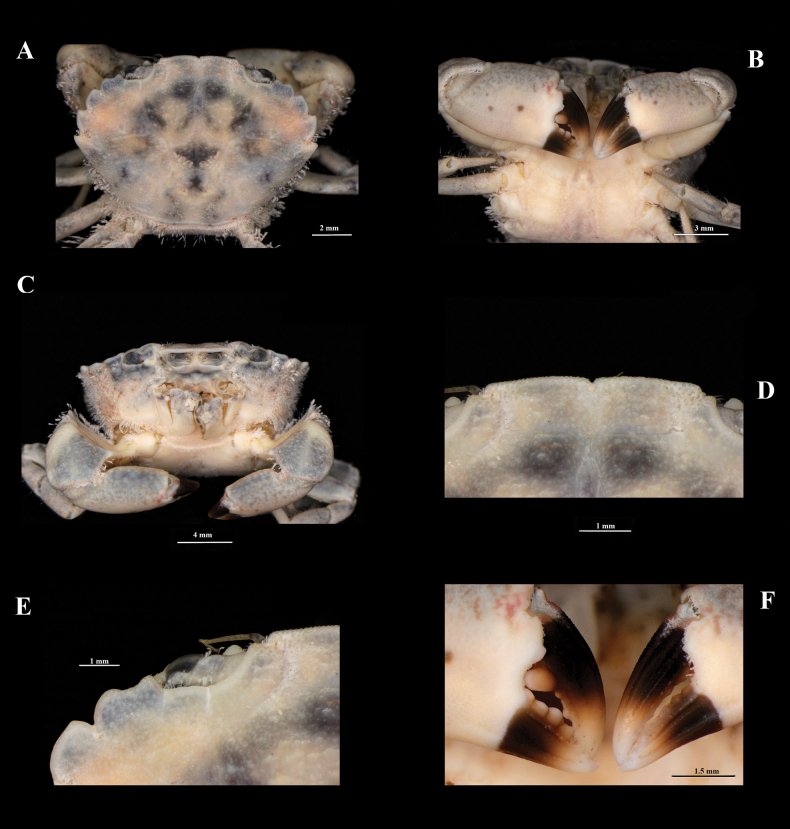
*Panopeuslacustris*. ECOSUR-C1197, male, 12.5 mm **A** body, dorsal view **B** body, dorsal view **C** frontal view **D** frontal margin **E** first two coalescent teeth **F** pollex and dactylus of chelipeds.

**Male**, ECOSUR-C1197, 12.5 mm (Fig. 4A–C, E). The male morphology is like the female, but there are still some differences. The color of the carapace is dark-brown with creamy-white areas.

The frontal margin is rectilinear (Fig. 4D). With a row of setae on the posterolateral and hind margins. Abdomen with five pereonites (the third, fourth and fifth pereonites fused), with a few small setae.

**Figure 5.**
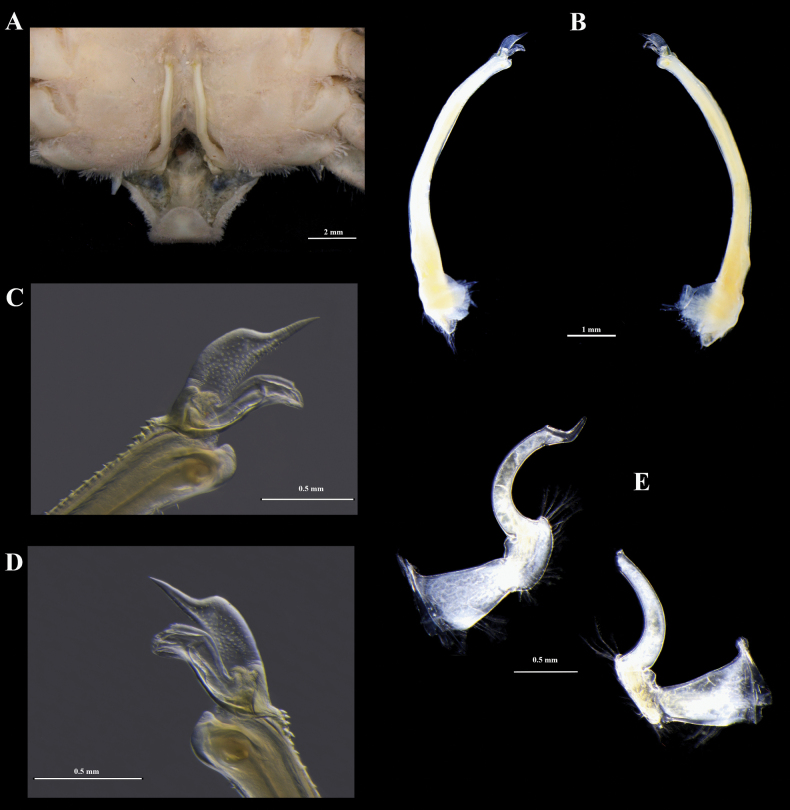
*Panopeuslacustris*. ECOSUR-C1197, male, 12.5 mm **A, B** gonopods 1 **C** detail of right gonopod 1 **D** detail of left gonopod 1 **E** gonopods 2.

The third pleonite with similar width but is shorter than the fourth pleonite; the fourth pleonite is shorter than the fifth pleonite; the fifth pleonite is twice as long as broad. Chelipeds (Fig. 4E), merus with similar length of the carpus; the reticulate pattern does not continue on its lower half, except in the minor chela, which slightly exceeds. Ambulatory legs are less setose than in females. Gonopod 1 (Fig. 5A–D), long and slender; slightly curving laterally; outer margin with two distal rows of conic spines that increase in thickness as they approach the anterior part of the outer margin; inner margin with six subdistal slender spinules. An accessory process long; dorsally expanded, with numerous ventrolateral spinules; anterior part narrow, ringed. The medial process is slightly sigmoidal, with subdistal spinules on the outer margin and robust proximal setae on the outer margin. Lateral approach bifid, with both teeth well developed. Gonopod 2 (Fig. 5E) is small, slightly sigmoidal.

#### Remarks.

Comparing morphological descriptions between specimens of *Panopeuslacustris* from Mexico, the USA, and the Virgin Islands, these were similar because they have a transverse line of granules on the carapace, the front margin of the carapace is granular and finely divided, the first two anterolateral carapace teeth are coalesced and separated by a round notch, the cheliped has broad proximal teeth on the pollex, and the pereopods are setose. Even so, there are some structural differences between them: *P.lacustris* from El Uvero, Mexico has a dark-brown or reddish-brown with creamy-white areas color pattern, while *P.lacustris* from Guadeloupe is dirty purple, and for Florida, USA, and St. Croix, VI the specimens are greyish; in *P.lacustris* from El Uvero, the carapace has some setae, whereas in *P.lacustris* from Guadeloupe, Florida, and St. Croix it is generally smooth. Table 2 provides more morphological comparisons.

##### ﻿Molecular analysis

**Figure 6.**
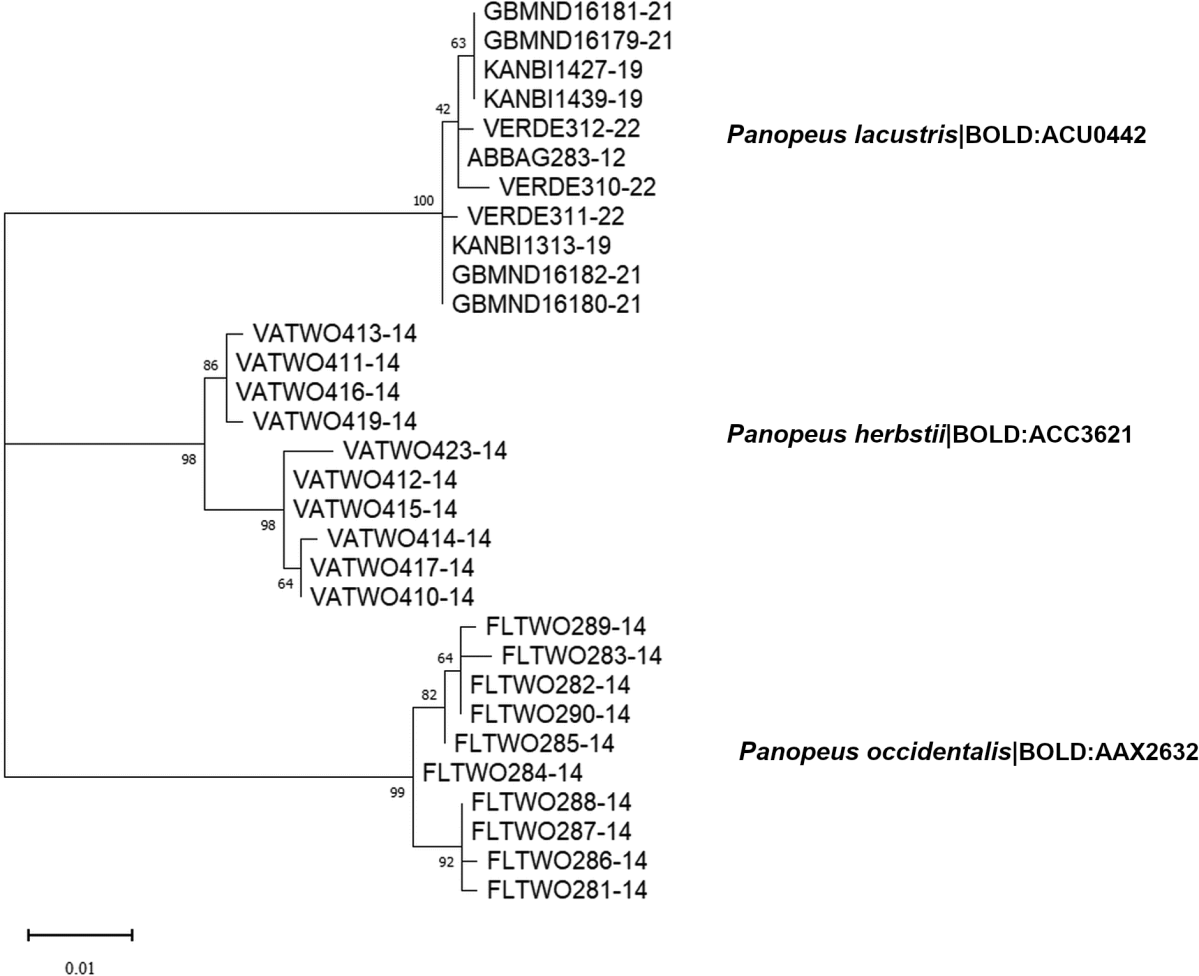
COI identification tree shows the clustering of *P.lacustris*, *P.herbstii* and *P.occidentalis* from the Southern Mexican Caribbean, Western Atlantic and Central Pacific. Numbers on the branches indicate bootstrap support after 500 replications. The numbers after the names are BINs.

Molecular analysis based on the COI gene for specimens of *P.lacustris* from the Southern Mexican Caribbean (El Uvero, Quintana Roo), Western Atlantic (Bocas del Toro, Panama), and Central Pacific (Hawaii, USA) shows that they belong to the same taxonomic identity because their intraspecific divergence value was 0.27% (*N* = 11), which according to Matzen da Silva et al. (2011) and Jarquín-González et al. (2022), remains below the range of intraspecific divergence (3.7–4.9%) reported in decapods. Additionally, *P.lacustris* showed an interspecific genetic variation of 5.78% with *P.herbstii* and 7.57% with *P.occidentalis*, confirming that they are distinct taxonomic entities (Fig. 6).

**Table 2.** Morphological comparison based on specimens of *P.lacustris* from the Western Atlantic.

	*P.lacustris* Current work	*P.lacustris* Desbonne 1867	*P.lacustris* Williams (1983)
Locality	El Uvero, Quintana Roo, Mexico	Guadeloupe, Antilles	Florida, USA and St Croix, Virgin Island
Carapace– Dorsal coloring pattern	Dark-brown or reddish-brown with creamy-white areas	Dirty purple	Grayish
Carapace– Setation	With few setae	Smooth or with few setae	Smooth
Carapace– Frontal margin	Granulose, with median closed fissure	Granulose, with median closed fissure	Granulose, with median closed fissure
Carapace–Transverse lines of granules	Present	Present	Present
Carapace– First and second anterolateral teeth	Coalesced	Coalesced	Coalesced
Carapace– Coalesced first and second anterolateral teeth	Separated by shallow rounded notch	-	Separated by deep rounded notch
Carapace–Tips of lateral teeth	Generally acute	-	Rectangular to acute
Third maxilliped– Spot color of ischium	Orange	-	Red
Cheliped–Palm: Reticulate pattern on outer surface continues over its lower half	No	-	Yes
Cheliped–Palm: Dark spots below the midline of the palm	Present	-	-
Cheliped–Pollex: Shape of proximal teeth	Broad	-	Broad
Cheliped–Dactylus: Proximal tooth shape	Subrectangular	-	Rounded
Pereopods–Setation	Setose	Setose	Setose

## ﻿Discussion

Historically, *Panopeus* has been recognized as a taxonomically complex and cryptic group (Williams 1983; Schubart et al. 2000; Thoma et al. 2014). This assumption is valid because, for 40 years, no new species have been recognized, no detailed updated taxonomic revisions have been made, and no complete updated taxonomic corrections have been made for this genus.

According to Rathbun (1930), Williams (1965, 1983), Spivak and Luppi (2005) and Marochi and Masunari (2011) identification of *Panopeus* species can be performed using morphological characteristics such as the anterolateral teeth of the carapace, the presence or absence of granules in the carapace and chelipeds, the presence and coloring of the spot on the ischium of the third maxilliped, the presence or absence of a reticulate pattern of coloration on the external surface of the chelipeds, and the teeth shape of the pollex in the mayor chela. However, these morphological characteristics only have been analyzed in a few species (e.g., *P.austrobesus*, *P.herbstii*, *P.lacustris*, *P.meridionalis*, *P.obesus* and *P.simpsoni*) of the 16 that make up the genus.

According to Martin and Abele (1986), the male gonopod is a beneficial morphological characteristic for separating species and genera within Panopeidae. Unfortunately, the morphology of this structure is not detailed or illustrated in most *Panopeus* species but only available for *P.africanus*, *P.americanus*, *P.austrobesus*, *P.chilensis*, *P.meridionalis* and *P.obesus* (Martin and Abele 1986; Spivak and Luppi 2005; Marochi and Masunari 2011; Thoma et al. 2014), and currently for *P.lacustris*. In this study it was possible to detect some morphological characteristics that could be useful in the recognition of the species, these are: the presence and quantity of chromatophores in mouthparts (e.g., first and second maxillipeds), and the ornamentation of the merus of cheliped (e.g., setae, spines, presence, or absence of spots); however, it is necessary to explore the morphological variation between and within species to corroborate its usefulness. To standardize and update the morphological information of the species of the genus *Panopeus*, it is recommended considering the morphological characters proposed by Williams (1983), Martin and Abele (1986), and this work.

Regarding molecular analysis, it is suggested to obtain genetic material for the species *P.boekei*, *P.convexus*, *P.diversus* and *P.meridionalis* since they do not have nuclear or mitochondrial sequences; complement the information associated with the genetic sequences of the species, including the collection site, type of habitat and photographs; and, resolve conflicts of identification for some COI sequences of the genus, for example, the species *P.americanus* presented two different BINs (BOLD:ADU0973, BOLD:ADX1970).

In addition, Thoma et al. (2014) mention that despite the species *P.herbstii*, *P.obesus* and *P.simpsoni* being currently considered different species (based on allozymes, hemocyanins, ecological and morphological differences), the available sequences of the mitochondrial (e.g., COI, 16S) gene do not help distinguish between them, probably because they have recently separated and have accumulated few mutations. Probably the same could be happening with the species *P.harttii* and *P.occidentalis* since they share the same BIN (BOLD:AAX2632).

It is fundamental to increase the knowledge of the gene pool of the group and the morphological characters that help separate species; these aspects will facilitate more detailed studies to detect general distribution patterns, link different life stages, and recognize cryptic species. With this, the taxonomic identification of *Panopeus* species will be precise.

## ﻿Conclusion

It is a priority that redescriptions of the species begin to be carried out, or provide detailed descriptions for male and female specimens, incorporate new morphological characteristics, and include genetic sequences. In this way, it will be possible to advance the taxonomic, and consequently other areas as ecological and conservation knowledge of the genus *Panopeus*. Furthermore, these results reinforce that studies with morphological and molecular approaches are useful for studying the biological diversity of marine and coastal decapods as previously detected for members of these group and allies.

## Supplementary Material

XML Treatment for Panopeus lacustris

## ﻿Additional information

### Author ORCIDs

Jani Jarquín-González https://orcid.org/0000-0002-8492-1725

Martha Valdez-Moreno https://orcid.org/0000-0001-5369-1667

Rigoberto Rosas-Luis https://orcid.org/0000-0002-7785-7120

### Data availability

All of the data that support the findings of this study are available in the main text.
